# Monitoring Carbon Ion Beams Transverse Position Detecting Charged Secondary Fragments: Results From Patient Treatment Performed at CNAO

**DOI:** 10.3389/fonc.2021.601784

**Published:** 2021-06-10

**Authors:** Marco Toppi, Guido Baroni, Giuseppe Battistoni, Maria Giuseppina Bisogni, Piergiorgio Cerello, Mario Ciocca, Patrizia De Maria, Micol De Simoni, Marco Donetti, Yunsheng Dong, Alessia Embriaco, Veronica Ferrero, Elisa Fiorina, Marta Fischetti, Gaia Franciosini, Aafke Christine Kraan, Carmela Luongo, Etesam Malekzadeh, Marco Magi, Carlo Mancini-Terracciano, Michela Marafini, Ilaria Mattei, Enrico Mazzoni, Riccardo Mirabelli, Alfredo Mirandola, Matteo Morrocchi, Silvia Muraro, Vincenzo Patera, Francesco Pennazio, Angelo Schiavi, Adalberto Sciubba, Elena Solfaroli-Camillocci, Giancarlo Sportelli, Sara Tampellini, Giacomo Traini, Serena Marta Valle, Barbara Vischioni, Viviana Vitolo, Alessio Sarti

**Affiliations:** ^1^ Dipartimento di Scienze di Base e Applicate per l’Ingegneria, Sapienza Università di Roma, Rome, Italy; ^2^ INFN Laboratori Nazionali di Frascati, Frascati, Italy; ^3^ Dipartimento di Elettronica Informazione e Bioingegneria, Politecnico di Milano, Milano, Italy; ^4^ INFN Section of Milan, Milan, Italy; ^5^ Dipartimento di Fisica “E. Fermi”, Università di Pisa, Pisa, Italy; ^6^ INFN Sezione di Pisa, Pisa, Italy; ^7^ INFN Sezione di Torino, Turin, Italy; ^8^ CNAO Centro Nazionale di Adroterapia Oncologica, Pavia, Italy; ^9^ Scuola di Specializzazione in Fisica Medica, Sapienza Università di Roma, Roma, Italy; ^10^ Dipartimento di Fisica, Sapienza Università di Roma, Rome, Italy; ^11^ INFN Section of Rome 1, Rome, Italy; ^12^ Dipartimento di Fisica, Università degli studi di Milano, Milan, Italy; ^13^ INFN Sezione di Pavia, Pavia, Italy; ^14^ Dipartimento di Chimica e Chimica Industriale, Università di Pisa, Pisa, Italy; ^15^ CREF - Museo Storico della Fisica e Centro Studi e Ricerche E.Fermi, Rome, Italy

**Keywords:** particle therapy, carbon ions, online monitoring, charged particles, fibre detectors

## Abstract

Particle therapy in which deep seated tumours are treated using ^12^C ions (Carbon Ions RadioTherapy or CIRT) exploits the high conformity in the dose release, the high relative biological effectiveness and low oxygen enhancement ratio of such projectiles. The advantages of CIRT are driving a rapid increase in the number of centres that are trying to implement such technique. To fully profit from the ballistic precision achievable in delivering the dose to the target volume an online range verification system would be needed, but currently missing. The ^12^C ions beams range could only be monitored by looking at the secondary radiation emitted by the primary beam interaction with the patient tissues and no technical solution capable of the needed precision has been adopted in the clinical centres yet. The detection of charged secondary fragments, mainly protons, emitted by the patient is a promising approach, and is currently being explored in clinical trials at CNAO. Charged particles are easy to detect and can be back-tracked to the emission point with high efficiency in an almost background-free environment. These fragments are the product of projectiles fragmentation, and are hence mainly produced along the beam path inside the patient. This experimental signature can be used to monitor the beam position in the plane orthogonal to its flight direction, providing an online feedback to the beam transverse position monitor chambers used in the clinical centres. This information could be used to cross-check, validate and calibrate, whenever needed, the information provided by the ion chambers already implemented in most clinical centres as beam control detectors. In this paper we study the feasibility of such strategy in the clinical routine, analysing the data collected during the clinical trial performed at the CNAO facility on patients treated using ^12^C ions and monitored using the Dose Profiler (DP) detector developed within the INSIDE project. On the basis of the data collected monitoring three patients, the technique potential and limitations will be discussed.

## Introduction

Carbon ion beams in Particle Therapy (PT) are used to achieve a high dose conformation to the target volume in combination with a high Relative Biological Effectiveness (RBE) (1). According to the Particle Therapy Co-operative group (PTCOG), thirteen ^12^C ions beam facilities located in Italy, Austria, Germany, China and Japan are currently in operation (2), and five are under construction. At present, a wide spectrum of pathologies located in several districts is eligible for carbon ion therapy. The reader is addressed to (3) for an updated review of the diseases treatable with carbon ions and the corresponding clinical outcome.

Despite the physical and biological advantages of carbon ion therapy, its intrinsic accuracy in targeting the treatment volume is not yet fully exploited. In the current clinical work-flow, most of the QA procedures are performed before the treatment, then all the arising inter-fraction effects as patient mis-alignment or morphological changes, which translate in an effective range difference with respect to the planning, have to be taken into account at the planning stage. A typical approach is the use of safety margins after defining the Clinical Target Volume (CTV) and safe irradiation strategies that avoid the potential exposure of organs at risk to unwanted dose (4, 5).

Great efforts have been made to develop a technique capable of giving a real time feedback on the dose conformity to the target volume. Such systems are typically based on the detection of secondary radiations as prompt-gammas (6), annihilation photons produced by the beam-induced *β* ^+^ activation (7, 8), or charged fragments (9, 10).

The Dose Profiler (DP) has been designed and built to be operated at CNAO as an *in vivo* verification system of the carbon ion treatments (11). It exploits charged secondary fragments, mainly protons, that are detected and tracked by means of eight planes of plastic scintillating fibers. The DP is a part of a bi-modal system, developed within the INSIDE collaboration (12) and installed in the CNAO treatment room n.1, including also a PET scanner used to measure the beam-induced *β* ^+^ activity. In 2019 a clinical trial started with the aim of evaluating the system capability to detect the morphological changes occurred in the patient during the several session of a full treatment delivery. The results obtained monitoring the first three patients can be found in Fischetti et al. (13), where the authors discuss the case of a patient for which internal morphological changes were detected by comparing the fragments spatial emission maps measured in different treatment fractions. In this manuscript, instead, we focus on a completely different matter: the possibility to exploit the secondary fragments produced during the treatment to monitor the beam position at the entrance point in the patient body. Such monitoring will be complementary to the techniques that are already routinely implemented in clinical centres to control the beam delivery and that are usually implemented using ionization chambers positioned at the end of the accelerator nozzle just before the beam exit window (14).

A CIRT treatment is composed of many irradiation by single Pencil Beams (PBs), with own scheduled direction, energy (i.e range) and fluency. Presently the transverse beam position of each PB is generally verified on-line by *ad hoc* devices [i.e. ionization chambers (15)] placed before the beam exit window. However, as stated in (16), a robust monitoring strategy independent of the diagnostics embedded in the nozzle could be of great interest, in particular in the frame of adaptive radio therapy using image guidance.

In CIRT, protons and neutrons are the most abundant products of the incoming beam fragmentation occurring inside the patient tissues (17) and a significant fraction of the protons produced at large angles with respect to the beam direction has enough kinetic energy to escape from the patient, as reported in several measurements (9, 18–20). In (16) a method based on the detection of such charged secondary fragments has been proposed, and its performance has been evaluated on an anthropomorphic phantom for different energies of the carbon ion beam. In this work we propose a monitoring technology, alternative to the ones currently implemented in the clinical centres using ionization chambers, based on charged fragments detection, and we evaluate its feasibility in the clinical practice analysing the data collected monitoring three patients enrolled in the INSIDE clinical trial.

The obtained results and the technique performance and limitations are reported and discussed hereafter.

## Material and Methods

Unlike neutral radiation, secondary charged particles can be easily detected and back-tracked with high efficiency and with little background. The measured fragments emission yield is anti-correlated with the production depth, since the kinetic energy of fragments decreases with the increasing path travelled inside the patient.

**Figure 1:**
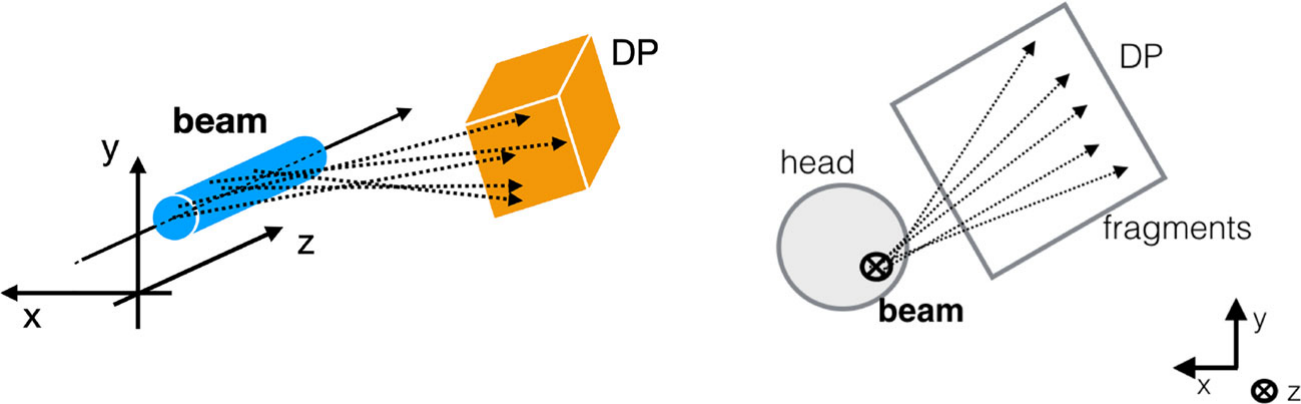
Sketch of the experimental setup. On the left a 3D sketch is showing the measurement principle: the production point of the fragments (dashed lines) detected by the DP are all located around the transverse beam position, within the beam lateral size (cyan cylinder). On the right the 2D projection is shown from the perspective in which the beam (black bold cross) is orthogonal to the picture. The rationale of the strategy proposed in the manuscript can be observed: in the plane orthogonal to the beam direction, the tracks intersections can be used to identify the beam incoming direction in the x,y plane.

Fragments that have the kinetic energy needed to exit from the patient are mainly products of the projectile fragmentation, as the products of target fragmentation have, in average, lower kinetic energies and are not able to exit from the body to be detected. In this latter case the products have kinetic energy of few MeV and can not escape from the patient, while projectiles fragments mainly keep the beam velocity and direction, causing the characteristic dose tail beyond the Bragg peak. The same arguments applies to the products of re-interactions of fragments inside the patient body (tertiary fragments): such fragments can be produced (especially in the case of neutrons) far away from the primary interaction of the beam with the patient along the path towards the target volume, but their contribution becomes to be significant only in the distal region where the direct production from the fragmentation drops. In the entrance channel, however, the fragments are mainly produced directly by the fragmentation of the projectile and for that reason their production vertexes have to lie in a truncated cone whose circular section, at different depths inside the patient body, has a radius that is a convolution of the beam spot size and the effect of the multiple scattering interactions undergone by the primary beam. The fragments produced at large angle (60°-90° with respect to the incoming beam direction) are mainly protons, with a low contamination of deuteron and tritons (less than 10%) and most of them are generated directly from the primary projectile fragmentation (21). When back-tracking those reconstructed fragments, towards their production region inside the patient, and performs the projection of the reconstructed tracks in the plane orthogonal to the beam direction, one thus expects an accumulation along the beam incoming direction with the aforementioned experimental uncertainty, as shown in **Figure 1**.

In this work, we therefore propose to evaluate the beam transverse position as the accumulation point in the plane orthogonal to the beam direction of the fragments-related tracks reconstructed by a tracking detector.

The method has been tested analysing the data collected monitoring three patients treated with carbon ions at CNAO. The data collection occurred in the context of the clinical trial at CNAO (22), as described in *Clinical Trial and Data Taking Conditions*, in which a test of the performance of the INSIDE system was carried out.

To evaluate the monitoring precision achievable on the incoming beam position, fragments coming from each PB were reconstructed, and their position in the transverse plane was compared to the nominal one provided by the Dose Delivery System (DDS). In the following, the details about the patient treatment and the tracking detector used for the monitoring are quickly summarized. The full procedure used to measure the beam position in the transverse plane is described in detail afterwards.

### The Dose Profiler

**Figure 2:**
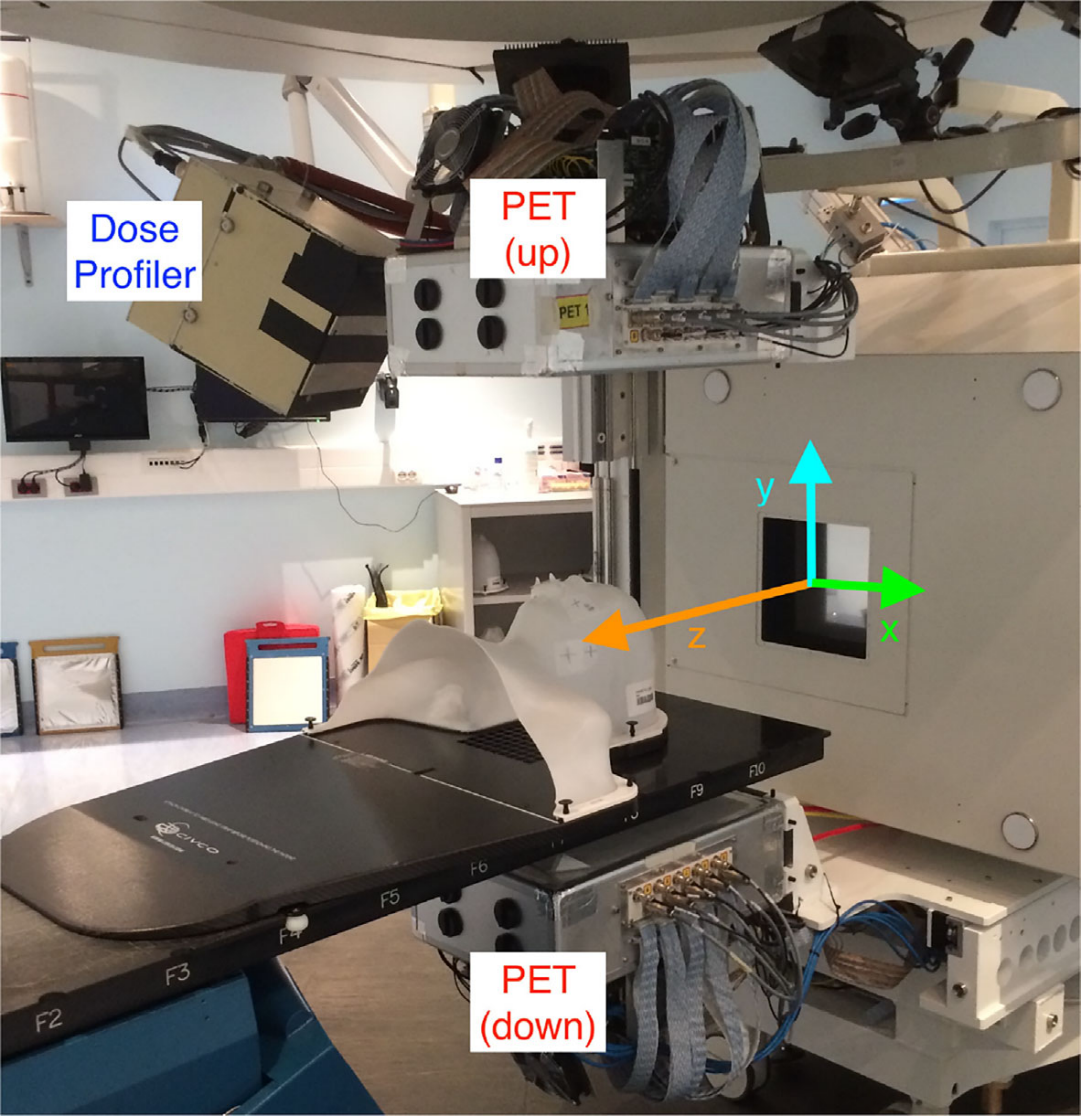
View of the INSIDE cart holding the DP and the PET heads installed in the CNAO treatment room 1. A patient mask is attached to the bed to show the patient position with respect to the DP during a treatment. The reference frame used to present the DP measurements is over-imposed (the *z*-axis, in orange, is along the incoming beam direction).

The DP [whose detailed description and performance can be found elsewhere (11)] is made of 8 scintillating fibers planes (each fiber has a square cross-section with 500 *μ*m side) and has been carefully optimized to detect and reconstruct the charged fragments exiting from the patient. More than 3,000 Silicon Photo-multipliers (each one of 1 mm^2^ active area) are used to collect the scintillation light from pairs of fibres in each plane and reconstruct the 3D path traversed by the fragments inside the detector active volume. The DAQ system, capable of collecting the signals from all the SiPMs and providing a self-triggering acquisition mode, was optimized to minimize the detector dead time (measured using the data collected during the patient monitoring and equal to ~5 *μ*s per event), allowing to sustain the fragment detection rate (*O* ~ 100 kHz) reached in a typical treatment at CNAO. A per track back-pointing resolution of 5–7 mm, depending on the fragment energy and angle inside the detector, has been measured with the device placed at 50 cm from a point-like target in a pre-trial characterisation data-taking campaign. The fiber planes and the read-out electronics are embedded in a light-tight box held by a movable cart (shown in **Figure 2**) that also support a PET scanner formed by two planar LYSO detectors, used to measure the beam-induced *β* ^+^ activation. The cart is inserted and hooked in the operation position just before the treatment start, and it is moved back to a rest position located in a room side once the treatment is finished.

During its operation the DP is located at ~50 cm from the room isocenter, forming an angle respectively of 60° with respect to the beam direction (z) in the xz plane, and of 30° in the yz plane. A precise measurement of the DP position with respect to the treatment room isocenter has been obtained by means of a laser survey system. It was found that the cart anchoring system allows for a highly reproducible positioning when removing and inserting the cart ensuring an accuracy of this procedure below 1 mm, as evaluated in the system commissioning phase.

### Clinical Trial and Data Taking Conditions

The INSIDE Clinical trial (22) has started in September 2019 at CNAO with the purpose of evaluating the carbon ion treatments inter-fraction monitoring capability of the DP. Ten patients, affected by pathologies involving the head-neck district, have been selected and monitored during the whole period of the therapy administration (3–4 weeks, typically four fractions per week). The clinical study was performed in accordance with all the relevant guidelines and running regulations on clinical trials and was approved by the referral ethics committee “CNAO” with the code CNAO-OSSINSIDE-02-18 on July 31, 2019; the informed consent was obtained from all the adult participants enrolled. No information or images that could lead to identification of the participant are present in this work.

We have analyzed the data of three patients affected by an Adenoid Cystic Carcinoma (ACC) of salivary glands, monitored during the clinical trial and already examined in Fischetti et al. (13) with the names PZA, PZB, and PZC. While the reader can found the full treatment plans description in the cited manuscript, the number of monitored treatment fraction, the number of delivered PB, the number of ions per PB as well as the beam energies foreseen by each plan are reported in **Table 1**. A Range Shifter (RS, a solid water 3 cm thick layer positioned between the beam exit window and the patient along the beam path) was used when delivering the treatment of all the considered patients.

**Table 1:** Details of the treatment plans delivered to the patients considered in this paper.

Patient ID	PZA	PZB	PZC
n. monitored fractions	6	10	6
n. PB	~ 37k	~ 7k	~ 33k
n. ions per PB	10^4^ - 8·10^5^	10^4^ - 1.5·10^5^	10^4^ - 7·10^5^
kinetic energies	126–297 MeV/u	153–269 MeV/u	126–278 MeV/u

### Transverse Position Assessment

The fragments position measurement starts from the signal registration performed for each triggered event. The fragments crossing the DP produce light in the scintillating fibers, which is detected by the SiPMs to build a 3D track inside the detector local reference frame using the Hough transform (23) applied to each detected ‘hit’. The track parameters are hence evaluated with a linear fit, as described in details in Traini et al. (11). The laser survey results are finally used to transform the track parameters in the global reference frame of the treatment room. While performing this change of reference frame the systematic uncertainty due to the DP positioning accuracy (at the level of 1 mm) is assumed to be negligible as the contribution from the limited statistics and multiple scattering on the final results are significantly larger. The high incoming fragment rate (more than 100 kHz in some of the slices that have to be treated with high energy and high number of ions) resulted in a significant fraction of events (~ 10%) with a track multiplicity larger than 1. Such events have been rejected to avoid the additional contribution to the position measurement uncertainty. Starting from the fully reconstructed sample, the tracks projections in the plane (xy), orthogonal to the beam direction, are computed. With such information, a 2D histogram representing the track density *ρ*_Track_ (*x*,*y*) in the transverse plane is built for each PB using the measured emission points along the beam path inside the patient of all the reconstructed tracks. A binning of 3 ×3 mm^2^, comparable with the CNAO carbon ion beam spot size (24), has been chosen. According to the MC simulation of the full treatment, performed with the FLUKA software (25, 26) and described in (13), the average angular deflection of the escaped fragments provoked by the multiple scattering is of the order of 60 mrad. For this reason the track density distributions do not present an evident peak for PBs with a low number of reconstructed tracks. We decided then to apply to each histogram a filter to avoid that the statistical fluctuations could result in a bias affecting the peak measured position. Different filters have been investigated: Gaussian, Median and Average based algorithms were applied to the 2D distribution and the measured PB positions have been compared with the nominal ones provided by the DDS. Among the different available filters we selected the Gaussian one, as it provided an unbiased result for all the data analysed. Different resolutions were tested, and the best results have been obtained smoothing the picture applying a 2D Gaussian filter with a *σ*_f_ of 1.0 cm.

**Figure 3:**
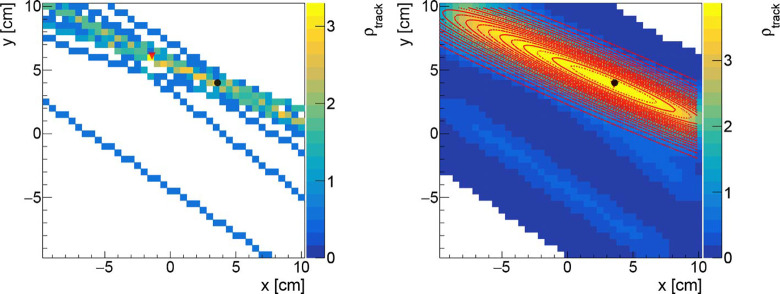
Left: Track density histogram for a PB with 17 reconstructed tracks (no filter applied). Right: the same figure is drawn applying a Gaussian filter with *σ*_f_ equal to 1.0 cm. Both figures are obtained with a binning of 3 mm in both axis. The black (circle) and the red (triangle) markers represents respectively the nominal beam position, and the reconstructed one. The 2D Gaussian fit is super-imposed (red curves).

An example of the track density histogram before and after the smoothing is shown in **Figure 3** respectively in the Left and Right panels. The observed stretched shape, asymmetrical in the vertical and horizontal axis, is due to the relative positioning of the DP with respect to the beam incoming direction. Since the DP is place at 60° with respect to the treatment room z axis, in the x,z plane, the resolution that can be obtained on the x position of the PCA is worse when compared to the one achievable along the vertical axis. This geometrical effect results in the shape that can be observed in **Figure 3**. A 2D elliptical Gaussian function was used to fit the data when estimating the distribution maximum value and measuring the PB position (x_meas_,y_meas_).

## Results

**Figure 4:**
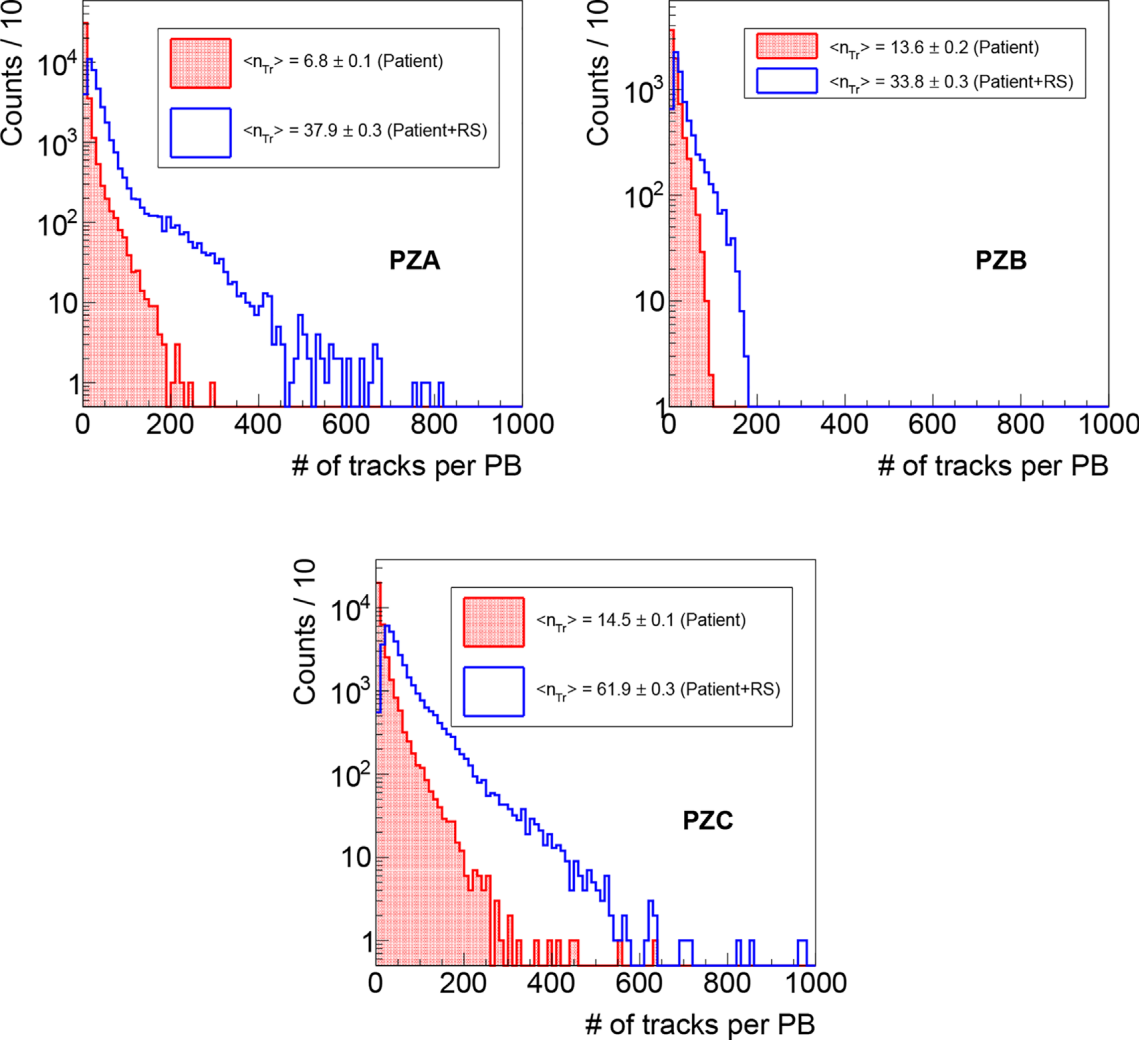
Distributions of the number of reconstructed tracks per PB as measured during the first fraction monitoring for PZA, PZB and PZC, obtained respectively selecting the fragments produced only in the patient (red line, dotted area), and that ones produced also in the RS (blue line, empty area).

To evaluate the precision and the accuracy of the method outlined in the previous section, for each PB the measured position (evaluated as the accumulation point position identified as explained in Section *Transverse Position Assessment* and shown in **Figure 3**) has been compared with the nominal one provided by the DDS (27), which unambiguously identifies the position of each PB in each treatment fraction. All the reconstructed tracks have a well defined DDS identifier and can be associated to a given PB. When considering the overall track sample, ~50–70% of the detected particles (depending on the patient positioning) are produced when the beam interacts with the range shifter, while the remaining ones are produced by the interaction with the patient. Despite that the former ones could be certainly used for the transverse position assessment, they have been excluded from this analysis in order to investigate the worst case scenario in which the treatment is performed without the RS and the fragments are emitted only by the patient. Applying such selection, the average number of reconstructed tracks per PB is ~7, ~14 and ~15 respectively for PZA, PZB and PZC, as can be observed in **Figure 4** where the distributions of the number of tracks measured in the first treatments fraction are shown as an example.

**Figure 5:**
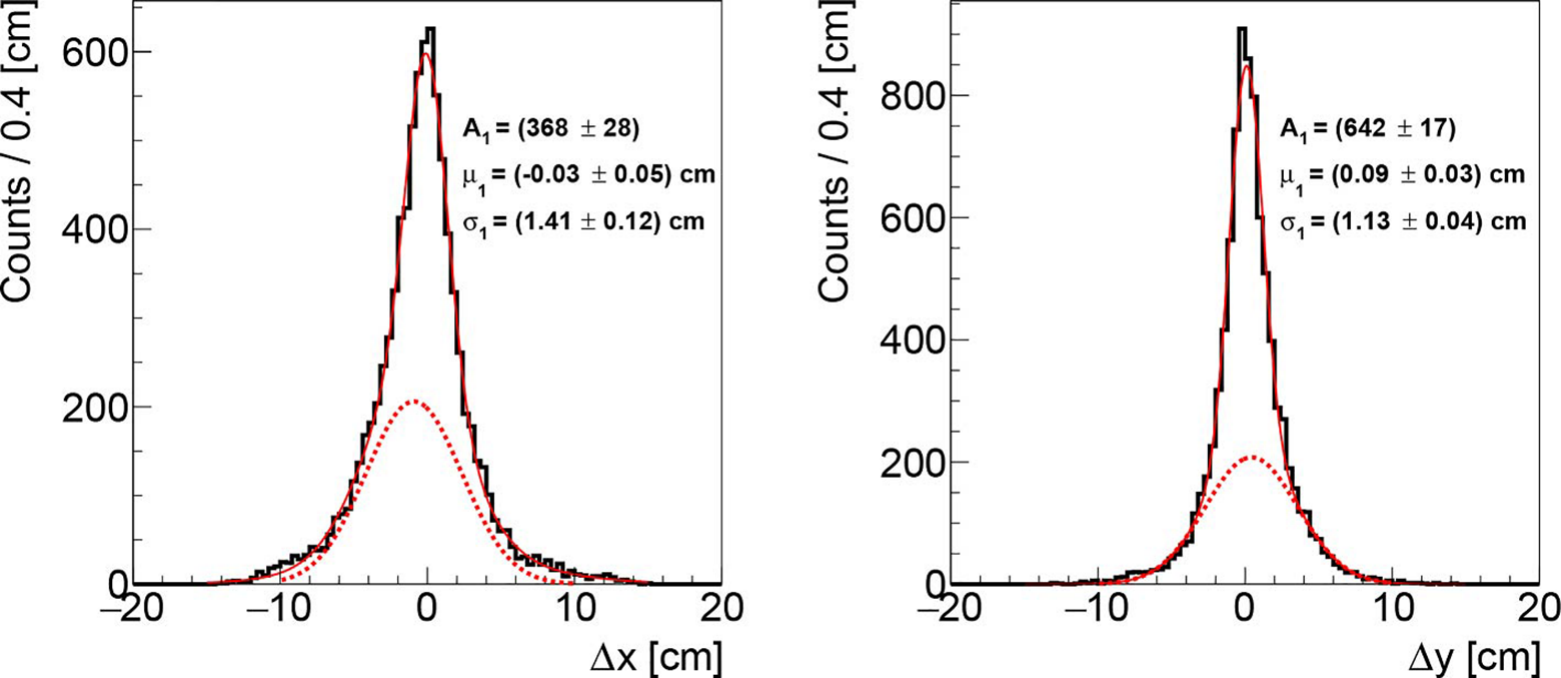
Distributions of the differences between the measured and the actual beam transverse position, respectively in the x (left) and y (right) axis, obtainded analysing the data acquired during the first fraction of PZC. The solid, red line represents the overall fit function while the dotted, red line highlights the tail contribution.

The distributions of the differences Δx = x_meas_ - x_nom_ and Δy = y_meas_ - y_nom_ of the reconstructed PB positions using the algorithms outlined here-before (x_meas_,y_meas_) with respect to the nominal PB ones (x_nom_,y_nom_) as coming from the raster file are shown in **Figure 5** for the first fraction of PZC.The histograms have been populated selecting only the PBs with a number of reconstructed tracks coming from the patient larger than 5 (~ 80% of the total number of PBs in the treatment fraction).

Both distributions show a Gaussian core (solid, red line) with a slightly asymmetrical tail (parametrized with a further Gaussian function with different central value shown as a dotted red line), due to the fact that the DP orientation is not orthogonal to the beam line, as described in section *The Dose Profiler*. The cores have respectively *σ_x_* ~ 1.4 cm and *σ_y_* ~ 1.1 cm along the x and y axes, while the fraction of the events associated to the tail is ~20%. The mean of the distributions is found to be consistent with zero (within a 1 mm bias that has a negligible impact on the results), confirming that the technique is able to follow the PB scanning without introducing a systematic uncertainty that has to be accounted for. Finally, the obtained results are not significantly affected by little variations of the filtering parameter *σ*_f_ (see section *Transverse Position Assessment*), as *σ_x_*, *σ_y_* vary of ±0.1 cm when using a *σ*_f_ between 0.8 and 1.2 cm.

Similar resolution have been obtained also for PZA and PZB. The results are very stable against the different treatment fractions, as summarized in **Table 2**, where the mean value <*σ_x_*>, <*σ_x_*> and the corresponding standard deviations $S_{\sigma_{x}},S_{\sigma_{y}}$, of the Gaussian core sigmas are reported for the three patients.

**Table 2:** Mean values <*σ_x_*>, <*σ_x_*> and standard deviations $S_{\sigma_{x}},s_{\sigma_{y}}$ of the resolutions obtained in the different treatment fraction for PZA, PZB and PZC.

Patient ID	PZA	PZB	PZC
n. monitored fractions	6	10	6
<*σ_x_*>	(1.55 ± 0.02) cm	(1.58 ± 0.03) cm	(1.41 ± 0.02) cm
<*σ_y_*>	(1.08 ± 0.02) cm	(1.09 ± 0.02) cm	(1.17 ± 0.02) cm
$S_{\sigma_{x}}$	0.05 cm	0.08 cm	0.04 cm
$S_{\sigma_{y}}$	0.03 cm	0.06 cm	0.05 cm

The measured resolutions shown in **Table 2** are significantly larger than the PBs spatial separation (2 mm) limiting the single PB monitoring capability of the DP. However, as stated in section *Transverse Position Assessment*, the accuracy on the transverse position is expected to be strongly slice and position dependent, as the number of reconstructed tracks per PB is highly affected by both the initial beam kinetic energy and by the amount of material that fragments have to cross to exit from the patient.

**Figure 6:**
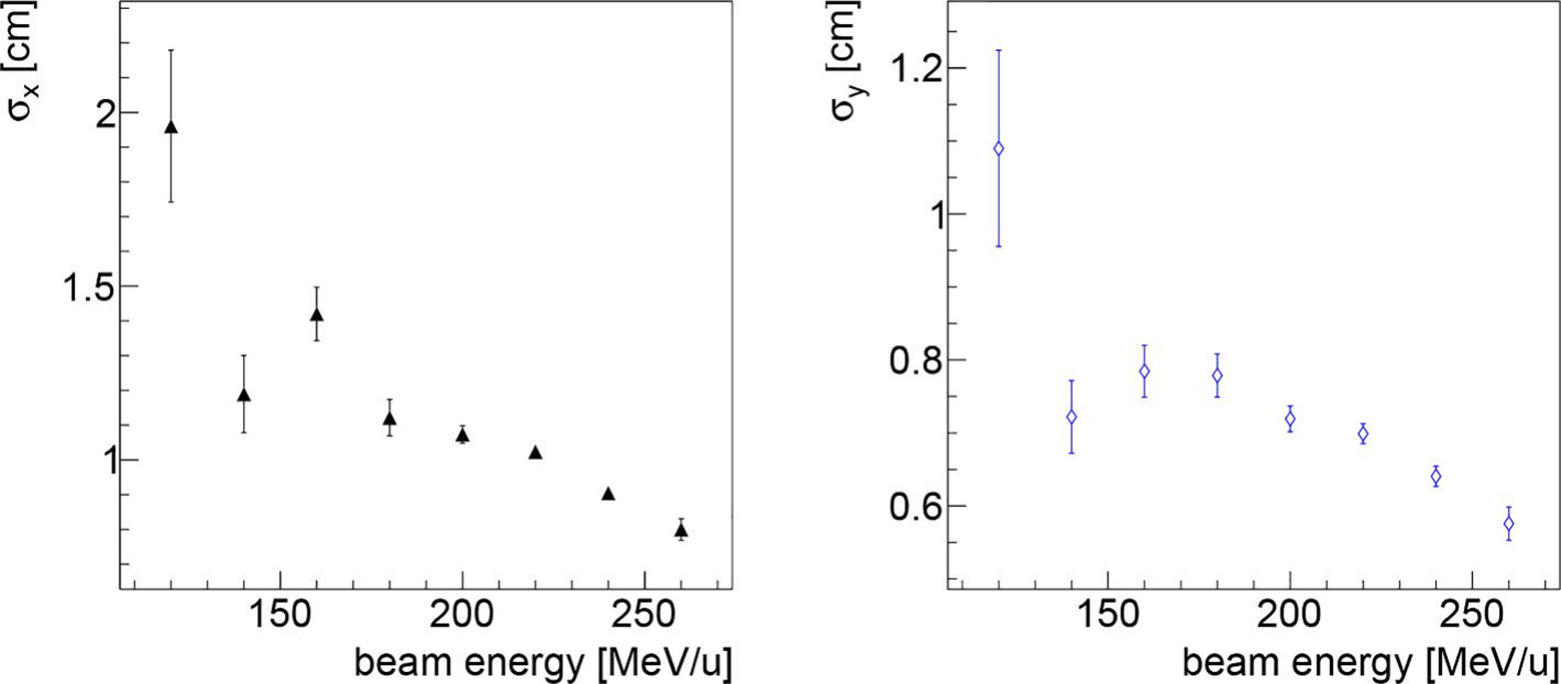
Resolution on the beam transverse position as a function of the beam energy along the x (left) and y (right) directions, obtained analysing the data collected in first fraction of PZC. As expected, the higher is the beam energy, the better is the resolution, as expected since there is a larger number of emitted fragments that are capable of escaping from the patient.

To study the potential of the technique assuming that the detector technology could be changed, the dependence of the obtained resolution on the beam energy and the collectable statistic has been studied using the data collected in the first fraction of PZC. In such analysis also the fragments produced in the RS have been included. The resolution dependence on the beam energy can be clearly observed in **Figure 6**. The observed behaviour is due to the larger number of fragments emitted when delivering PB with higher energies.

**Figure 7:**
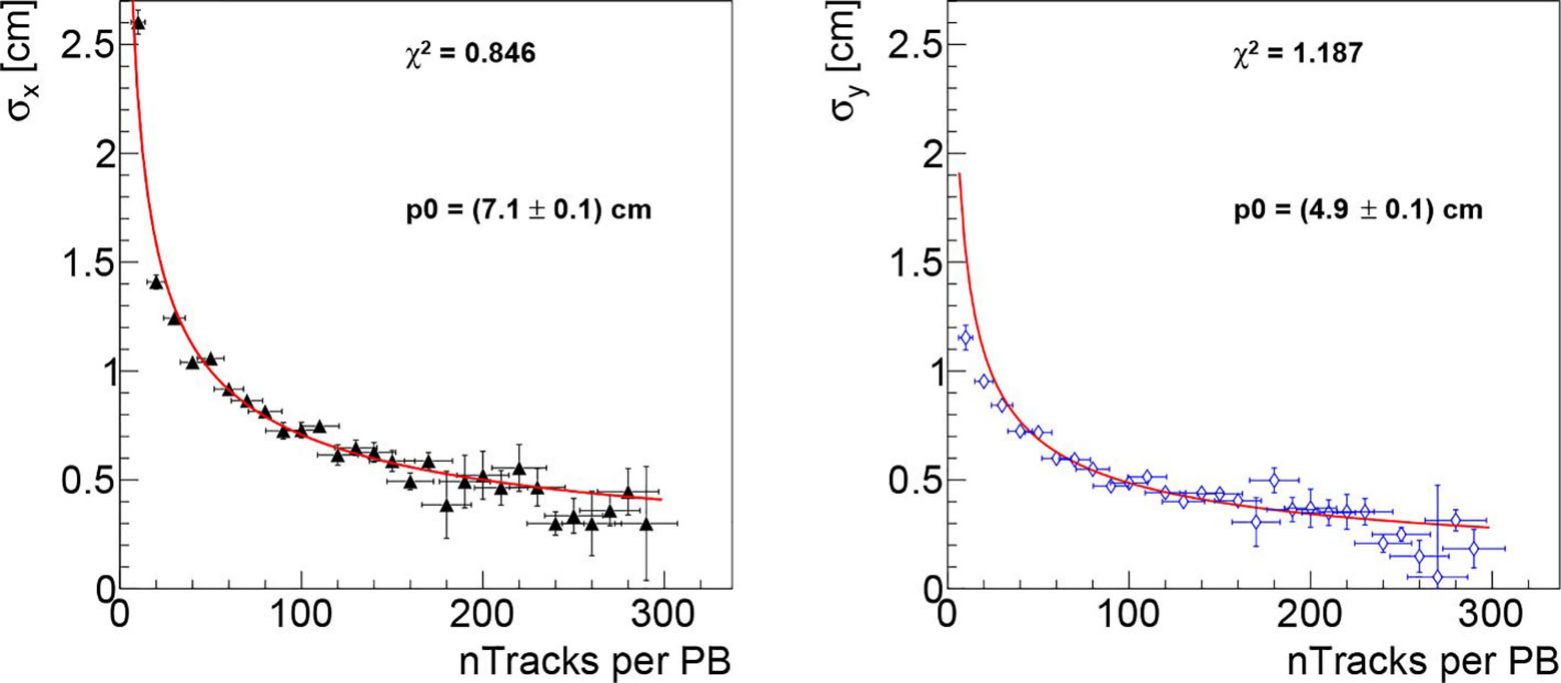
Beam transverse position resolution as a function of the number of reconstructed tracks per PB, respectively for the x (left) and y (right) directions, obtained analysing the data collected in first fraction of PZC. As expected, the resolution scales proportionally to $p_{0}/\sqrt{N}$, the trend is superimposed on the figures in red to guide the eye.

The dependence on the collectable statistics is shown in **Figure 7** where the x and y position resolutions are shown as a function of the number of collected tracks per PB. The resolution scales as expected, following the $p_{0}/\sqrt{N}$ trend which is over-imposed on both plots to guide the eye.

Concerning the DP monitoring capabilities in a real case scenario, an average number of tracks per PB <n_Tr_> between 5 and 15 and between 35 and 60 is observed respectively when selecting only the particles produced within the patient and when considering also the ones produced in the RS, as shown in **Figure 4**.

## Discussion

In this manuscript we explored, in the frame of CIRT technology, the online monitoring capabilities of the beam transverse position using a charged fragments detector. The real case of three patients treated for an ACC was used to collect the data and evaluate the performance in a clinical scenario. The reported results suggest that the accuracy of such technique is mainly limited by two factors:

- the multiple scattering suffered by the fragments travelling within the patient from their production point towards the detector, which add an unavoidable resolution term to the measurement of the accumulation point of the reconstructed tracks;
- the number of particles detected per PB. The number of reconstructed tracks per PB is, on average, few tens, with a strong dependency on the patient treatment details and on the energy of the incoming projectiles. In order to improve the resolution, strategies to increase the number of detectable fragments have to be defined and implemented.

**Figure 7** shows that the resolution decreases, in the range up to 300 tracks per PB, as $1/\sqrt{N}$, and we can therefore use the observed behaviour to predict the expected resolution for larger number of tracks. To reach a resolution comparable with the lateral PB distance (2 mm), a number of tracks per PB >500 would be needed according to the $1/\sqrt{N}$ scaling and the DP absolute positioning in the room reference frame would need to be known with a better precision (smaller than the current uncertainty of: 1 mm). In that case an absolute position resolution measurement, performed online, could be used to provide a valuable independent feedback to the DDS and to the treatment QA software. With lower number of tracks the resolution degrades and only the average position of close PB will be accessible.

The number of fragments that can be detected by the dose profiler while monitoring a CIRT treatment is limited by the detector dead time (: 5 µs at the measured DAQ rates (~ 60-70 kHz in average with peaks above 100 kHz). While the presence of RS can significantly boost the number of detectable fragments, we have shown that very few PBs could match the >500 requirement even if these additional tracks are considered.

We estimate that reducing the detector dead time, the detectable fragments could be easily doubled. To reach the required precision, we are still missing a factor ~ 15 (worst case scenario) and ~4 (if all available tracks coming either from the patient or from the RS can be used) in statistics: a possible solution might be to enlarge the detector acceptance (increasing the active volume or putting the detector closer to the patient) or to reduce the tilt angle with respect to the beam line, at the expense of some additional distortion effect when back-projecting the tracks.

These changes might not be easy to implement in the current setup of the INSIDE system. Thus, to confirm the capability of the proposed technique of monitoring of the transverse beam position in carbon ions treatments with a resolution comparable or lower the lateral PB spatial separation, as suggested by the data trend, an adequate technological solution capable of overcoming the current DP limitations will be needed.

## Data Availability Statement

The raw data supporting the conclusions of this article will be made available by the authors, without undue reservation.

## Ethics Statement

The studies involving human participants were reviewed and approved by CNAO (code:CNAO-OSSINSIDE-02-18). The patients/participants provided their written informed consent to participate in this study.

## Author Contributions

GT, MT, and ASar wrote the main manuscript text and prepared the figures. GBat, GBis, PC, MS, YD, AE, VF, EF, AK, EM, MMor, SM, FP, GT, SV, and ASar took active part in acquiring the data during the INSIDE clinical trial. GBar, MC, MD, CL, MMag, EMaz, AM, GS, ST, BV, and VV played a decisive role in setting up the INSIDE cart, the INSIDE infrastructure and starting and selecting the patients for the clinical trial. MS, IM, MMar, VP, ASar, ASci, ASch, and GT have proposed the idea of the Dose Profiler detector, supervised the detector operation and the data taking. PM, MS, MF, GF, VP, ASar, ASci, ASch, GT, and MT implemented and performed the data analysis. All authors contributed to the article and approved the submitted version.

## Conflict of Interest

The authors declare that the research was conducted in the absence of any commercial or financial relationships that could be construed as a potential conflict of interest.
